# DUSP6, a tumor suppressor, is involved in differentiation and apoptosis in esophageal squamous cell carcinoma

**DOI:** 10.3892/ol.2013.1605

**Published:** 2013-10-07

**Authors:** JIANJUAN MA, XIYING YU, LIPING GUO, SHIH HSIN LU

**Affiliations:** State Key Laboratory of Molecular Oncology and Department of Etiology and Carcinogenesis, Cancer Institute and Hospital, Chinese Academy of Medical Sciences and Peking Union Medical College, Beijing 100021, P.R. China

**Keywords:** dual-specificity phosphatase 6, differentiation, apoptosis, methylation, esophageal squamous cell carcinoma

## Abstract

Dual-specificity phosphatase 6 (DUSP6), a specific negative feedback regulator of phosphorylated extracellular signal-regulated kinase, was found to play an important role in numerous types of solid tumors as a tumor suppressor. In this study, 64.2% (61/95) of esophageal squamous cell carcinoma (ESCC) specimens studied exhibited reduced DUSP6 protein expression, compared with 91% (81/89) of normal esophageal specimens that displayed moderate or strong DUSP6 protein expression in tissue microarray analysis. In total, 36.8% (7/19) of the tumor biopsies displayed at least two-fold downregulation of DUSP6 compared with their paired normal counterparts, by qPCR. Significant loss of DUSP6 was observed in EC9706 and KYSE150 ESCC cell lines by immunoblotting assay. Low DUSP6 protein expression was significantly associated with pathological grade in ESCC by immunohistochemistry (P<0.05). Treatment with 5-aza-2′-deoxycytidine restored DUSP6 expression in the two ESCC cell lines, and the expression varied according to the drug concentration. Methylation-specific PCR analysis showed methylation-specific products in the two ESCC cell lines. We observed significant differences in the early and total apoptotic proportion between the control and experimental groups of the two ESCC cell lines and their transfectants (P<0.001) by annexin/propidium iodide assay. The presence of cleaved PARP product, a marker of caspase-mediated apoptosis, expressed in the two pCMV-DUSP6 transfectants in marked contrast to the parental and pCMV-transfected EC9706 and KYSE150 cells, was observed by immunoblotting. Overall, our results support the role of DUSP6 as a novel candidate tumor suppressor gene in ESCC, which may be a potential prognostic marker for ESCC.

## Introduction

Dual specificity phosphatase 6 (DUSP6) is a mitogen-activated protein kinase (MAPK) phosphatase that plays a critical role as a negative regulator of the MAPK pathway (1,2). The MAPK pathway controls a vast array of physiological processes, including cell proliferation, cell cycle arrest, cell survival and cell motility (3–6). Specifically, DUSP6 dephosphorylates the threonine and tyrosine residues of extracellular signal-regulated kinase (ERK) 1/2, and inactivates ERK1/2 in a feedback loop (7–9). Disruption of this feedback loop may give rise to increased exposure to growth factors, resulting in neoplastic or even malignant transformation (2,10,11).

In different types of tumors, DUSP6 have various roles depending on the tumor type and the stage of carcinogenesis. Previous studies have indicated that DUSP6 acts as a tumor suppressor gene in several types of tumors (12–23). In pancreatic cancer, the expression of DUSP6 was downregulated, which activated ERK excessively, and eventually led to improvement of the carcinoma development and progression (12–14). The downregulation of DUSP6 was caused by the hypermethylation of CpG sequences in intron 1 of the DUSP6 gene in the progression of pancreatic cancer (15). The DUSP6 expression correlated inversely with the growth activity and histological grade of the tumor in lung cancer (16). In ovarian cancer, DUSP6 expression is lost, particularly at the protein level, leading to the hyperactivation of ERK1/2 and eventually resulting in tumorigenicity and chemoresistance of human ovarian cancer cells (17). Notably, DUSP6 has a contrary effect in certain other tumor types. DUSP6 is upregulated in myeloma (18), melanoma (19), glioma (20), glioblastoma (21), keratinocytes (22) and breast cancer (23). In glioblastoma, the overexpression of DUSP6 lessens tumor cell sensitivity to the anticancer DNA-damaging drug cisplatin (21). Overexpression of DUSP6 causes estrogen receptor-positive breast cancer cells to become resistant to the growth inhibitory effects of tamoxifen (23). Furthermore, methylation of DUSP6 is infrequent in endometrial cancer (24). Therefore, silencing of DUSP6 may not be involved in the constitutive activation of the ERK kinase cascade in endometrial cancer (24).

However, few studies have reported the role of DUSP6 in esophageal cancer. Esophageal squamous cell carcinoma (ESCC) is a potentially fatal disease with high incidence worldwide, particularly in China (25). Despite recent progress in ESCC diagnosis and treatment, the survival rates for ESCC patients remain poor. Thus, there is a requirement for studying new genes involved in ESCC tumorigenesis and progression, in order to develop safer and faster diagnosis and improved disease outcome predication following treatment of this dangerous disease.

A previous investigation showed downregulation of DUSP6 in ESCC in Hong Kong (26). Furthermore, our previous study demonstrated that the exogenous overexpression of DUSP6 confirmed the growth suppression in ESCC cells (27). In the present study, we focus on the correlation between DUSP6 expression and clinicopathological features, the mechanisms by which DUSP6 affects the ESCC cells and the possible epigenetic mechanisms involved in the abrogation of DUSP6 in ESCC cells.

## Materials and methods

### Cell lines and primary tissues

The 19 paired ESCC and normal esophageal tissues were obtained from patients who underwent surgery at Henan Cancer Hospital (Zhengzhou, China). The tissue microarrays were purchased from Biomax (Rockville, MD, USA; ES1202 and ES8010). The arrays included a total of 89 benign esophageal tissue samples and 95 localized esophageal cancer samples. Two human esophageal squamous cell carcinoma cell lines, EC9706 and KYSE150, were used in this study. The EC9706 cell line was established and studied by Han *et al*(28), while the KYSE150 cell line was kindly provided by Dr Shimada (First Department of Surgery, Faculty of Medicine, Kyoto University, Japan). The two cell lines were cultured in accordance with their original methods (28,29). The present study was approved by the institutional review boards of the Cancer Institute and Hospital, Chinese Academy of Medical Sciences and Peking Union Medical College (Beijing, China). Written informed consent was obtained from the patients.

### Cellular transfection

The empty vector (pCMV-AC) and the plasmid containing the complementary DNA sequence of human DUSP6 (pCMV-DUSP6) were purchased from OriGene (Beijing, China). The ESCC cells, EC9706 and KYSE150, were transiently transfected with pCMV-AC or pCMV-DUSP6, using Lipofectamine™ 2000 according to the manufacturer’s instructions (Invitrogen Life Technologies, Carlsbad, CA, USA). Whole cell lysates for immunoblotting were collected at 24 h after transfection in order to confirm the appropriate plasmid DUSP6 expression.

### qPCR

The total RNA collected from each sample was extracted using TRIzol reagent according to manufacturer’s instructions (Invitrogen Life Technologies). Total RNA was then used to synthesize cDNA using PrimeScript Rtase (Takara, Shiga, Japan). The RT product was used as the template to amplify DUSP6. The forward and reverse primers were 5′-AAC AGG GTT CCA GCA CAG CAG-3′ and 5′-GGC CAG ACA CAT TCC AGC AA-3′, respectively. GAPDH was used as an internal control. The products were resolved by electrophoresis in 3% agar and stained with ethidium bromide. DUSP6 expression levels were evaluated by qPCR using StepOne RealTime PCR system (Applied Biosystems, Beijing, China). The data were normalized by the intensity of GAPDH.

### Western blotting

Western blotting was performed as described previously (30). The cells were lysed in 1% NP-40 lysis buffer and cleared by centrifugation at 12,000 × g for 20 min. Supernatants were recovered as protein extracts. The extracts containing equal amount of proteins were separated using 10% sodium dodecyl sulfate-polyacrylamide gel electrophoresis and transferred to polyvinylidene difluoride membranes (Millipore, Billerica, MA, USA). The membranes were incubated with non-fat dry milk in 0.01 M/l Tris-buffered saline containing 0.1% Tween-20 to block non-immunospecific protein binding, and then with primary antibody against DUSP6 (OriGene) and phosphorylated ERK (p-ERK; Santa Cruz Biotechnology, Santa Cruz, CA, USA). Following incubation with a peroxidase-conjugated affinipure goat anti-rabbit IgG secondary antibody (Jackson Immuno Research, West Grove, PA, USA), protein signals were visualized by enhanced chemiluminescence (Pierce, Rockford, IL USA).

### Immunohistochemistry

Formalin-fixed tissue sections (4 μm) were deparaffinized and rehydrated, and incubated with 3% hydrogen peroxide, followed by antigen retrieval treatment, boiling in citrate buffer (0.01 M/l, pH 6.0), to restore the masked epitope. Goat serum (10%) was used to block endogenous peroxidase activities and non-immunospecific protein binding. The sections were then incubated with primary antibody against DUSP6 (OriGene) overnight at 4°C. After washing with phosphate-buffered saline (PBS) with 1% Tween-20, the sections were incubated with secondary horseradish peroxidase-conjugated antibody for 30 min at room temperature. Immunoreactivity was visualized with freshly prepared diaminobenzidine substrate and the nuclei were counterstained with hematoxylin.

DUSP6 expression levels in esophageal cancer cells were subdivided into four categories, negative (0), faint (1), moderate (2) and strong (3). Negative (0) was defined as tissues with no staining. Faint (1) expression was defined as tissues with faint staining or moderate to strong staining in <25% of cells. Moderate (2) was defined as a moderate or strong staining in 25–50% of cells. Strong (3) was defined as a strong staining in >50% of cells. The cut-off point to define high and low DUSP6 expression was 25% staining.

### Treatment with 5-aza-2′-deoxycytidine

The ESCC cell lines were first cultured in 10-cm plates and the cells were maintained until they reached 30% confluence. Subsequently, the cells were treated with 10 μmol/l of 5-aza-2′-deoxycytidine (Sigma, St. Louis, MO, USA) for five days, as described previously (16). These cells were harvested for further investigation.

### Methylation-specific PCR assays

As it was reported that the region in intron 1 of the DUSP6 gene is highly methylated in pancreatic cancer (15), we designed primer sets for both unmethylated (forward: 5′-GTA GGG GTT GTG AAT TGT GT-3′ and reverse: 5′-AC CAC CAA TAC CCA CAA CCA-3′) and methylated (forward: 5′-GTA GGG GTC GCG AAT CGC GC-3′ and reverse 5′-ACC GCC GAT ACC CGC AAC CG-3′) sequences at the highest methylated region in intron 1 of DUSP6. PCR conditions were as previously described (15).

### Annexin/propidium iodide assays

After the EC9706 and KYSE150 cells were transiently transfected with plasmid constructs, the two cell lines and their transfectants were collected by trypsinization and washed with PBS, and subsequently stained with annexin/PI. Samples were analyzed by FACScan flow cytometry (Becton Dickinson, Franklin Lakes, NJ, USA), according to the manufacturer’s instructions.

### Statistical analysis

All statistical analyses was performed with the SPSS software (version 17.0; SPSS Inc., Shanghai, China). Differences in DUSP6 protein expression between normal and ESCC specimens, and associations between DUSP6 expression and clinicopathological characteristics of ESCC patients, were analyzed by the χ^2^ test. The association between DUSP6 expression and pathological grade of ESCC patients in the tissue microarray assay was analyzed by Spearman’s rank correlation analysis. Student’s two-sided t-test was used to compare values of test and control samples. P<0.05 was considered to indicate a statistically significant difference. Results of the experiments are depicted as the means ± SD.

## Results

### DUSP6 is downregulated in ESCC

**Figure 1:**
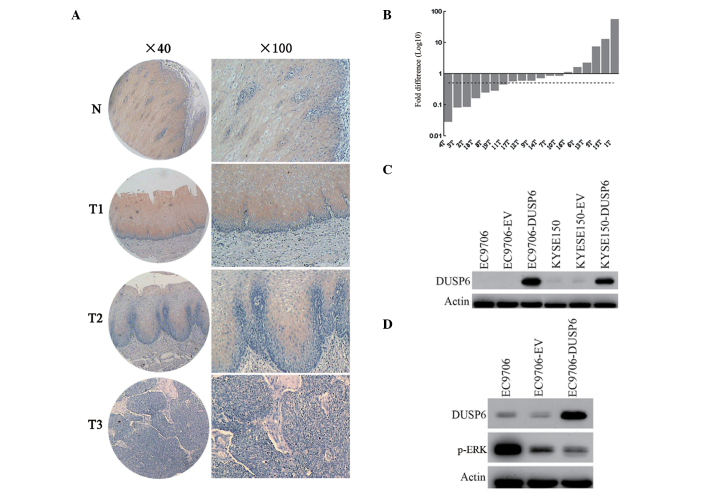
DUSP6 expression in esophageal cancer. (A) Expression of DUSP6 in normal esophageal epithelia and primary ESCC tumors were examined by immunohistochemistry. Normal esophageal epithelia are shown in panel N and the primary esophageal cancers are shown in panels T1, T2, and T3 (magnification: Left, ×40; right, ×100). (B) Expression of DUSP6 in paired normal and tumor tissues from the same patients by qPCR. In total, 36.8% (7/19) of the biopsies displayed at least two-fold downregulation of DUSP6 compared with their corresponding normal counterparts. The dotted line is shown to indicate the two-fold threshold of downregulation. (C) Western blot analysis was used to determine the DUSP6 expression in EC9706, empty vector-transfected EC9706, pCMV-DUSP6 (DUSP6)-transfected EC9706, KYSE150, empty vector-transfected KYSE150 and pCMV-DUSP6-transfected KYSE150 cells. (D) Western blot analysis was utilized to examine the DUSP6 and p-ERK expression in EC9706, empty vector-transfected EC9706 and pCMV-DUSP6 (DUSP6)-transfected EC9706 cells. DUSP6, dual-specificity phosphatase 6; ESCC, esophageal squamous cell carcinoma; p-ERK, phosphorylated extracellular signal-regulated kinase.

In this study, DUSP6 expression was examined at the mRNA and protein levels in ESCC. The DUSP6 protein expression level was measured by ESCC tissue microarray immunohistochemical staining (Fig. 1C). DUSP6 protein was predominantly observed in the cytoplasm. It was found that normal esophageal epithelia showed moderate or strong positive staining, yet cancer tissues demonstrated negative or weak immunoreactions. In total, 81 of 89 (91%) normal esophageal specimens studied showed DUSP6 protein expression and 61 of 95 (64.2%) ESCC specimens studied exhibited reduced DUSP6 protein expression. Statistical analysis revealed significant differences in DUSP6 protein expression between normal and ESCC specimens (P<0.000). qPCR was performed to evaluate the mRNA level of DUSP6 in 19 paired normal and tumor tissues from the same patients (Fig. 1A). In total, 68.4% (13/19) of the tumor biopsies expressed lower levels of DUSP6 than their corresponding normal counterparts; and 36.8% (7/19) of the tumor biopsies displayed at least two-fold downregulation of DUSP6 compared with their paired normal counterparts. The DUSP6 protein level in ESCC cell lines and their transfectants was further evaluated by western blotting (Fig. 1B). The results revealed that the two ESCC cell lines (EC9706 and KYSE150) and their pCMV transfectants exhibited extremely low DUSP6 protein expression, while their pCMV-DUSP6 transfectants showed markedly high DUSP6 protein expression. These results indicate that downregulated DUSP6 expression may be important in the tumorigenesis of ESCC.

It is widely accepted that DUSP6 is a negative feedback regulator in the MAPK pathway that acts by dephosphorylating the activated ERK (1). In the present study, DUSP6 and phosphorylated ERK protein expression were detected in the EC9706 ESCC cell line and its transfectants using western blotting. As shown in Fig. 1D, p-ERK expression decreased, while DUSP6 expression increased in parental, pCMV-AC transfected and pCMV-DUSP6 transfected EC9706 cells. Forced expression of DUSP6 in EC9706 significantly suppressed the p-ERK expression, indicating that increased DUSP6 expression is associated with downregulation of p-ERK *in vitro* in ESCC.

### Correlation between DUSP6 expression and clinicopathological features

We further analyzed the association between DUSP6 protein expression and clinicopathological features in ESCC (Table I). The age, gender and primary tumor size showed no significant correlations with the expression of DUSP6. Notably, significant correlations were observed between DUSP6 expression and pathological grade in ESCC (r=−0.257, P=0.015). It was also found that upregulated expression of DUSP6 was significantly correlated with regional lymph node metastasis. We speculate that this observation may be caused by the negative feedback loop of p-ERK to the tumorigenic signaling during lymph node metastasis.

### Promoter hypermethylation suppresses DUSP6 expression

**Figure 2:**
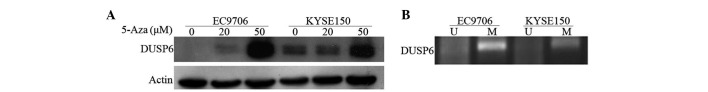
Methylation status of the DUSP6 promoter and intron 1. (A) Western blot analysis shows restoration of DUSP6 expression in two ESCC (EC9706 and KYSE150) cell lines after demethylation treatment by 5-aza-2′-deoxycytidine at two different concentrations (20 and 50 μM). Restored DUSP6 expression in both ESCC cell lines varied according to the 5-aza-2′-deoxycytidine concentration. (B) Methylation-specific PCR analysis: The region from +544 to +627 in intron 1 of the DUPS6 gene was PCR-amplified with primers specific for either methylated or unmethylated DNA as a template. The products were resolved by electrophoresis in 3% agar and stained with ethidium bromide. DUSP6, dual-specificity phosphatase 6; ESCC, esophageal squamous cell carcinoma; 5-Aza, 5-aza-2′-deoxycytidine; U, unmethylated; M, methylated.

In the present study, we evaluated the role of hypermethylation in the transcriptional suppression of DUSP6. The two ESCC cell lines (EC9706 and KYSE150) were treated with DNA methyl-transferase inhibitor 5-aza-2′-deoxycytidine at two different concentrations (20 and 50 μM). The restored DUSP6 expression was upregulated when the drug concentration was higher, as shown in Fig. 2A. The results indicated that the hypermethylation of the promoter was important in pathological suppression of DUSP6 transcription in ESCCs.

In order to determine whether hypermethylation occurs in the expressional regulatory regions of DUSP6, we performed methylation-specific PCR analysis. The high methylation of the region between +544 and +627 in intron 1 of DUSP6 was reported, accounting for the expressional suppression of DUSP6 in pancreatic cancer that was shown previously (15). Therefore, the present study focused on this region. The same methylation-specific PCR analysis was employed as described previously (15). Methylation-specific products in both ESCC cell lines (EC9706 and KYSE150) were observed, as expected (Fig. 2B).

In this case, it was demonstrated that the hypermethylation of CpG islands in intron 1 may be one main mechanism leading to the silencing of DUSP6 in esophageal squamous cancers.

### DUSP6 expression promotes apoptosis in ESCC

The functional effect of overexpressed DUSP6 on cellular apoptosis in EC9706 and KYSE150 cells transfected with the DUSP6 gene was examined.

**Figure 3:**
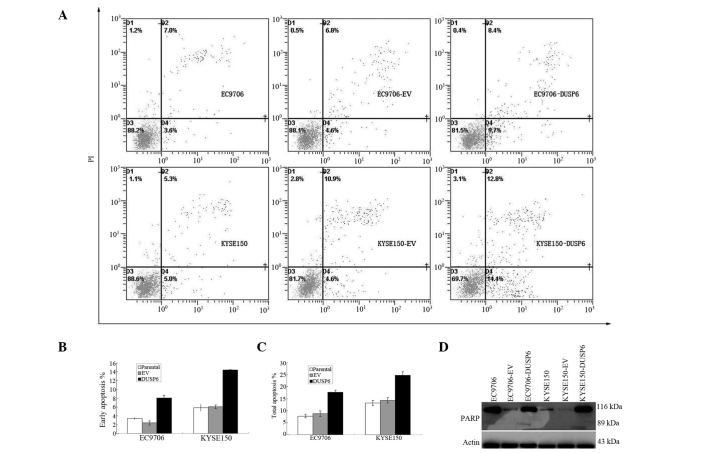
DUSP6 overexpression increased ESCC cell apoptosis via the PARP pathway. (A–C) Annexin/PI assay: EC9706 and KYSE150 cells were transiently transfected with either EV or pCMV-DUSP6 plasmids (DUSP6), stained with Annexin V-FITC and PI, then analyzed by flow cytometry. Results with percentage of cells listed for each quadrant: Lower left, viable cells; lower right, cells in early apoptosis; upper right, cells in late apoptosis. (A) Representative flow histograms. (B) Bar chart showing percentage of cells in early apoptosis by two cell lines and their transfectants. (C) Bar chart showing percentage of cells in total apoptosis by two cell lines and their transfectants. (D) PARP assay: Apoptosis in DUSP6-overexpressing EC9706 and KYSE150 transfectants confirmed by immunoblot detection of the 89-kDa PARP cleavage product. DUSP6, dual-specificity phosphatase 6; ESCC, esophageal squamous cell carcinoma; PI, propidium iodide; EV, empty vector; FITC, fluorescein isothiocyanate.

Following transfection with plasmids, the cells were stained with annexin V-FITC and PI to analyze the proportion of early and total apoptotic cells in both ESCC cell lines and their transfectants (Fig. 3A). It was found that both pCMV-DUSP6 transfectants displayed a marked increased in early and total apoptosis by annexin/PI assay. The mean early apoptotic cell proportion was 3.40±0.28, 2.40±0.46 and 8.05±0.56% in parental, pCMV-AC-transfected and pCMV-DUSP6-transfected EC9706 cells, respectively, and was 5.85±0.79, 6.08±0.41 and 14.45±0.05% in parental, pCMV-AC-transfected, and pCMV-DUSP6-transfected KYSE150 cells, respectively (Fig. 3B).

The mean total apoptotic cell proportion was 7.78±0.76, 8.8±1.21 and 17.68±0.0.99% in parental, pCMV-AC-transfected and pCMV-DUSP6-transfected EC9706 cells, respectively, and was 13.2±1.08, 14.3±1.10 and 24.73±1.45% in parental, pCMV-AC-transfected and pCMV-DUSP6-transfected KYSE150 cells, respectively (Fig. 3C). Statistical analysis showed significant differences in early and total apoptotic proportion between the control and experimental groups of the two ESCC cell lines and their transfectants (P<0.001).

Subsequently, cellular expression of PARP and its cleaved product was assayed by immunoblotting in the two ESCC cell lines and their transfectants. The presence of cleaved PARP product, a marker of caspase-mediated apoptosis, was found to be expressed in both pCMV-DUSP6 transfectants, in marked contrast to the parental and pCMV transfected EC9706 and KYSE150 cells, further confirming the induction of apoptosis by DUSP6 expression in these cells (Fig. 3D).

## Discussion

DUSP6 is an exclusive negative feedback regulator of activated ERK during normal development (1,31). In the present study, it was revealed that DUSP6 is a candidate tumor suppressor gene in ESCC. Initially, the ESCC cell lines and primary tumor specimens were screened, demonstrating that the DUSP6 expression level was downregulated at the mRNA and protein levels in ESCC. Using Spearman’s rank correlation analysis, DUSP6 expression was observed to be negatively correlated to pathological grade, indicating that DUSP6 is important in human ESCC carcinogenesis, particularly in tumor progression and differentiation. Subsequently, we demonstrated that the promoter hypermethylation accounted for the frequent low expression of DUSP6. Conversely, demethylation treatment restored DUSP6 expression in ESCC cells. Finally, we revealed that exogenous DUSP6 expression in both ESCC cell lines significantly induced apoptosis. Overall, these results implied that DUSP6 may serve as a tumor suppressor gene in ESCC, and loss of DUSP6 may be important in ESCC tumorigenesis.

DUSP6, one of the DUSPs family, is a highly selective phosphatase for ERK that appears to play a crucial role in development and the pathology of various diseases. Accumulating studies have demonstrated that DUSP6 is involved in tumor progression and resistance. In various types of cancer, DUSP6 acts in a contradictory manner. In pancreatic cancer, the DUSP6 gene was not identified to be expressed in the vast majority of pancreatic cancer cell lines and invasive primary pancreatic cancer tissues (12,13). DUSP6 exerts apparent tumor suppressive effects and is a strong candidate for a tumor suppressor gene (13,32). Consistent with our findings, a frequent loss of DUSP6 expression was observed as the histological grade of the tumor increased in lung cancer. DUSP6 is a potential tumor suppressor gene of lung cancer (16). However, DUSP6 is overexpressed and acts as an oncogene in certain other types of cancer (21,23). In the present study, we evaluated the expression at the mRNA and protein levels in ESCC cell lines and primary tumor specimens. It was demonstrated that DUSP6 was downregulated in ESCC. Tissue microarray assays indicated that DUSP6 expression inversely correlated with the histological grade, which suggested that lower DUSP6 expression was involved in tumor progression and differentiation. We hypothesized that DUSP6 may be a promising prognostic biomarker in ESCC. Further studies are required to validate the clinical utility of DUSP6 protein as a biomarker for ESCC prognosis.

The crucial mechanisms inactivating tumor suppressor genes are gene promoter hypermethylation, coding exon mutation and loss of heterozygosity (LOH). Previous studies have demonstrated that DUSP6 was downregulated by hypermethylation of intron 1 (12,15), LOH (16) or ubiquitination/proteasome degradation (17). In ESCC, tumor suppressor genes, such as FHIT, ECRG4 and DIRAS1, are downregulated by gene promoter hypermethylation (30,33,34). In the current study, our preliminary investigation of the DUSP6 downregulation focused on the epigenetic gene silencing, which is important in the initiation and progression of cancer (35,36). It was observed that methylation-specific products in ESCC cell lines were produced by methylation-specific PCR. Further pharmocological demethylation treatment restored the DUSP6 expression. These results indicated that the promoter hypermethylation may be one important factor resulting in the loss of DUSP6 expression in ESCC. Consistent with our results, a previous study showed that hypermethylation was also pivotal in downregulation of DUSP6 in ESCC in Hong Kong (37).

Functionally, DUSP6 has demonstrated suppressive effects in tumor formation and cancer cell mobility in ESCC in previous studies (27,37). DUSP6 played an important role in inducing cellular apoptosis in a previous study; the downregulation of DUSP6 mRNA by nitric oxide exerted antiapoptotic effects in endothelial cells (38). The exogenous expression of DUSP6/MKP-3 has been shown to induce apoptosis by the attenuation of ERK activation in pancreatic and lung cancer (13,39). In the present study, we investigated the effect of DUSP6 on cellular apoptosis in ESCC. Consistent with a previous study (39), our data showed that exogenous DUSP6 expression markedly increased early and total apoptosis *in vitro*, further supporting the fact that DUSP6 may be an important candidate tumor suppressor gene in ESCC.

It has been reported that the activation of the MEK/Erk pathway may inhibit cellular apoptosis (40–42) and that the inhibition of ERK is crucial for the induction of apoptosis (43). Given the established role of ERK1/2 in the antiapoptotic defense network and the fact that DUSP6 induced apoptosis and ERK was downregulated *in vitro* in ESCC, we speculated that the apoptotic effect of DUSP6 in ESCC may be induced by attenuation of ERK activation. More comprehensive investigations are required to determine the associations between DUSP6, ERK and apoptosis.

In conclusion, the current study has suggested that DUSP6 expression decreases with the depth of invasion in ESCC, predicting tumor progression independent of tumor grade. Enforced DUSP6 expression induced cellular apoptosis *in vitro* in ESCC. We hope that our data may provide new evidence to improve the understanding of the carcinogenesis of ESCC and that it will help to provide new therapeutic strategies for the treatment of ESCC.

## Figures and Tables

**Table I:** Association between DUSP6 expression and clinicopathological features in ESCC.

	DUSP6 staining, n (%)			
		
Variables	−	+	++	Correlation (P-value)
Age, years				
<60	21 (37.5)	30 (53.6)	5 (8.9)	0.114 (0.275)
≥60	13 (34.2)	16 (42.1)	9 (23.7)	
Gender				
Male	22 (34.9)	33 (52.4)	8 (12.7)	0.009 (0.930)
Female	12 (38.7)	13 (41.9)	6 (19.4)	
TNM classification				
pT				
pT_1_	3 (60)	2 (40)	0 (0)	0.170 (0.104)
pT_2_	10 (40)	13 (52)	2 (8)	
pT_3_	20 (31.7)	31 (49.2)	12 (19.0)	
N				
N_0_	33 (39.8)	40 (48.2)	10 (12.0)	0.253 (0.014)
N_1_	1 (10.0)	5 (50.0)	4 (40.0)	
N_2_	0 (0.0)	1 (100.0)	0 (0.0)	
Grade				
G_1_	2 (15.4)	8 (61.5)	3 (23.1)	−0.257 (0.015)
G_2_	12 (27.9)	24 (55.8)	7 (16.3)	
G_3_	17 (51.5)	12 (36.4)	4 (15.7)	

DUSP6, dual-specificity phosphatase 6; ESCC, esophageal squamous cell carcinoma.
